# S04-2: Government-driven physical activity policy monitoring across 27 countries: The EU HEPA Monitoring Framework

**DOI:** 10.1093/eurpub/ckae114.212

**Published:** 2024-09-26

**Authors:** Wanda Wendel-Vos

**Affiliations:** National Institute for Public Health and the Environment, Bilthoven, The Netherlands

## Abstract

**Purpose:**

National policy action is essential to increase population-level physical activity. To support these activities, the European Commission published Physical Activity Guidelines in 2008. In 2013, a Recommendation by the Council of the EU endorsed their implementation across Member States and called on governments to monitor their progress using a joint framework.

**Methods:**

The Council Recommendation established 23 indicators based on the categories of the EU Physical Activity Guidelines. A survey tool was developed with support from WHO Europe, accompanied by a Staff Working Document with background information and detailed instructions. An EU Physical Activity Focal Points Network was established in 2014 to support governments and ensure harmonized data collection across countries.

**Results:**

Supported by the European Commission and WHO Europe, EU Member States have conducted three rounds of data collection for the Monitoring Framework since 2015, with a fourth round in 2024. Results were published in summary documents and individual Country Factsheets in 2015, 2018 and 2021, as well as thematic factsheets and scientific publications. In a 2020 survey, a broad majority of Focal Points stated that the information was useful for national policy development, e.g. to gain insights from other countries, to communicate with national policymakers, and to exchange with other sectors.

**Conclusions:**

With 23 indicators for 27 countries, the EU HEPA Monitoring Framework is arguably the most comprehensive dataset currently available comparing national physical activity policy in a harmonized way. It is also one of the few examples for government-driven physical activity policy monitoring. The framework has proven to be an important tool for policy development in the EU and inspired data collection in several other countries. Future challenges include further developing the Framework to account for global challenges like climate change and for glass ceiling effects as national physical activity policies continue to evolve.

**Support/Funding Source:**

Data are collected by EU Member States. The WHO Regional Office for Europe provides support for data collection and analysis as via grant from the European Commission.

